# Determinants of Health Insurance Coverage among People Aged 45 and over in China: Who Buys Public, Private and Multiple Insurance

**DOI:** 10.1371/journal.pone.0161774

**Published:** 2016-08-26

**Authors:** Yinzi Jin, Zhiyuan Hou, Donglan Zhang

**Affiliations:** 1 China Center for Health Development Studies, Peking University. 38 Xue Yuan Road, Haidian District, Beijing 100191, China; 2 Department of Social Medicine, School of Public Health, National Key Laboratory of Health Technology Assessment (Ministry of Health), Collaborative Innovation Center of Social Risks Governance in Health, Fudan University, 138 Yi Xue Yuan Road, Shanghai 200032, China; 3 Department of Health Policy and Management, College of Public Health, University of Georgia, 100 Foster Road, Wright Hall, Athens, GA, United States of America; Old Dominion University, UNITED STATES

## Abstract

**Background:**

China is reforming and restructuring its health insurance system to achieve the goal of universal coverage. This study aims to understand the determinants of public, private and multiple insurance coverage among people of retirement-age in China.

**Methods:**

We used data from the China Health and Retirement Longitudinal Survey 2011 and 2013, a nationally representative survey of Chinese people aged 45 and over. Multinomial logit regression was performed to identify the determinants of public, private and multiple health insurance coverage. We also conducted logit regression to examine the association between public insurance coverage and demand for private insurance.

**Results:**

In 2013, 94.5% of this population had at least one type of public insurance, and 12.2% purchased private insurance. In general, we found that rural residents were less likely to be uninsured (Relative Risk Ratio (RRR) = 0.40, 95% Confidence Interval (CI): 0.34–0.47) and were less likely to buy private insurance (RRR = 0.22, 95% CI: 0.16–0.31). But rural-to-urban migrants were more likely to be uninsured (RRR = 1.39, 95% CI: 1.24–1.57). Public health insurance coverage may crowd out private insurance market (Odds Ratio = 0.55, 95% CI: 0.48–0.63), particularly among enrollees of Urban Resident Basic Medical Insurance. There exists a huge socioeconomic disparity in both public and private insurance coverage.

**Conclusion:**

The migrants, the poor and the vulnerable remained in the edge of the system. The growing private insurance market did not provide sufficient financial protection and did not cover the people with the greatest need. To achieve universal coverage and reduce socioeconomic disparity, China should integrate the urban and rural public insurance schemes across regions and remove the barriers for the middle-income and low-income to access private insurance.

## Introduction

China’s public health insurance system covers the largest number of population in the world [1], however to be precise, the system itself is fragmented rather than integrated. At present, there are three major types of public insurance—the New Rural Cooperative Medical Scheme (NCMS), the Urban Employee Basic Medical Insurance (UEBMI), and the Urban Resident Basic Medical Insurance (URBMI), covering 95% of the entire population in China [2]. The three insurance is designed according to the permanent residence registration system (aka the “Hukou” system) and/or the person’s employment status. The Hukou System classifies people as rural or urban residents based on their places of birth, which is not easily transferable from rural to urban residence [3]. For example, due to the Hukou System, rural people migrating and living in urban areas may still remain in their rural residence status. The three insurance targets different populations, and Hukou becomes the main barrier to shifting across these insurance plans.

- NCMS, targeting the registered rural population, is a “semi-mandatory” health insurance scheme. It was established in 2003 and administered by the Ministry of Health (currently the State Health and Family Planning Commission of China). NCMS pools its fund at the county level in the rural areas, whereby each rural county can design the benefit package and implementation plan, following the guidelines issued by the national and provincial governments.[4]
- UEBMI, targeting the urban employees, is legally mandatory that all employers must provide medical insurance to their employees. It was transformed from the previous labor medical insurance during China’s economic reform in 1990s.[5]
- URBMI, targeting the urban non-employee residents including children and adolescents under age 18, is a voluntary health insurance scheme that has been established since 2007 [6]. The UEBMI and URBMI are both administered by the Ministry of Human Resources and Social Security. UEBMI and URBMI pool their funds at the municipal level in the urban areas.[7]

The three public insurance varies significantly in the amount of premiums, benefit packages and reimbursement rates for different health care services. Among the three insurance, UEBMI offers the most generous benefit packages, while NCMS is often considered the most rudimentary type of insurance [8]. In 2013, the annual premium of NCMS and URBMI was about 350 Chinese Renminbi (RMB) per capita, but the annual premium of UEBMI reached 2500 RMB per capita [9]. NCMS only covers 800–1200 types of medications, but UEBMI and URBMI cover more than 2100 types of medications, almost doubled the coverage of NCMS. UEBMI and NCMS reimburse both outpatient and inpatient care, whereas URBMI mainly provides reimbursement for inpatient care [10]. The reimbursement rates for inpatient care reached 50.1%, 53.6% and 68.8% respectively for NCMS, URBMI and UEBMI in 2013 [11]. However, preventive care services and luxury medical services such as high technologies, cosmetic and plastic surgery, as well as services covered by other insurance schemes (e.g., injury protection insurance and maternity insurance) are not reimbursed through any of the three public insurance schemes [7].

There are large variations in the economic development and population size across the rural counties and urban cities. Lack of integration among these three schemes results in non-portability across insurance schemes and geographic areas, which have left certain population without insurance and some with multiple insurance coverage [2, 12]. In particular, a growing number of migrant workers are rushing into urban cities from rural regions [13]. Since public health insurance is pooled and administered at local county- or municipal- level, insurance agencies often designate local health care facilities as their coverage network; in other words, health services received out of the local county or city are possibly not reimbursed by local public insurance. Thus, migrants have incentives to shift their insurance or participate in multiple insurance to obtain reimbursement. Although those rural-to-urban migrants are eligible for both rural and urban public health insurance, in 2014, about 14.9% of migrants did not have any health insurance [14]. But at the same time, some migrants were covered by multiple insurance. A previous study showed that, from 2007 to 2010, 2.9% of migrant workers had been covered by two types of insurance, among whom 1.5% enrolled in NCMS and URBMI, and 1.2% in NCMS and UEBMI [15]. Given that there were 253 million migrants in 2014, it is estimated that 7.3 million have repeatedly enrolled in public health insurance and get duplicated financial subsidies from governments [15, 16]. Therefore, it is important to understand the determinants of insurance coverage particularly among the migrant population. Nevertheless, there has been no research focusing on this issue in China.

Another research gap is about the demand for private health insurance. Although public health insurance has covered the majority of population in mainland China, it only offers limited financial protection [17]. While with the rising challenges of chronic diseases [18], private health insurance plays an increasingly critical role to fill the coverage gap and meet the various health care needs of the population [19]. Since formally recognized and regulated by the Chinese government in 1998, private health insurance has remained slow in its development [20]. In 2009, China initiated a national healthcare system reform, and one of the reformed policies was to promote the development of private health insurance. The Chinese government encouraged the insurance companies to provide various insurance products, especially for the elderly, disabled and those with catastrophic diseases, and it also encouraged individuals to purchase these insurance products. During the healthcare reform, various strategies have been released in order to promote its development, such as the exemption of business tax for private insurers, preferential tax rate of health insurance for individuals and allowing UEBMI enrollees to purchase private health insurance with individual medical savings accounts [21]. Private insurers were also allowed to manage the public health insurance plans. It is reported that there were about 100 private insurers offering over 1500 health insurance products in 2012 [17]. The products mainly covered catastrophic diseases with little reimbursement for services like outpatient or nursing home care [22]. The premium from private health insurance had increased from 3.6 billion to 69.2 billion RMB from 1999 to 2011, and its share of the total health care expenditures also increased from 0.9% to 3.4% [23]. Private health insurance is available in both rural and urban China, targeting mainly the high-income population. According to the National Health Services Survey [11], 6.9% of the Chinese population purchased private health insurance in 2013. During the period of 2008 to 2013, the proportion of the population who purchased private insurance had increased from 6.9% to 7.7% in urban areas, but decreased from 6.9% to 6.1% in rural areas. While most studies to date focused on public health insurance in China, few literature has ever examined the private health insurance coverage as well as the dual coverage of public and private health insurance [22]. This paper is among the first to investigate demands of private health insurance among the population of retirement age in China.

The present study examined the determinants of public, private and multiple insurance coverage among the Chinese people aged 45 and older. We used the China Health and Retirement Longitudinal Study (CHARLS) to answer two research questions: 1) what are the characteristics of those who were covered by public, private, multiple urban and rural public health insurance, as well as private and public health insurance? And 2) what is the association between public insurance coverage and the demand for private health insurance?

## Methods

### Study design and data

We used the 2011 and 2013 panel of the CHARLS data, a nationally representative sample of Chinese people aged 45 and over, and their spouses [24]. It was modeled after the Health and Retirement Study in the United States, covering questions on demographics, socioeconomic status, health insurance, and health status and health behaviors. Age 45 was selected as the cutoff age by the CHARLS was because it is the minimum retirement age (the minimum age for receiving pension) in China [25]. We were particularly interested in this middle-aged and elderly population because with the rising prevalence of chronic diseases among this population, they had a higher demand for health insurance [26]. The details of the data have been described elsewhere previously [27]. Ethical consent was approved by the Institutional Review Board of Peking University.

The CHARLS national sample was drawn using the stratified four-stage cluster sampling method. In the first sampling stage, 150 county-level units (rural counties or urban districts) were randomly selected by Probability Proportional to Size (PPS) from a sampling frame containing all county-level units of mainland China excluding those in Tibet. Within each county-level unit, 3 primary sampling units (PSUs)–administrative villages in rural areas or neighborhoods in urban areas–were randomly selected by PPS. Within each PSU, 24 households with members aged 45 or older were randomly selected. The member aged 45 or older and his or her spouse (if present) were interviewed face-to-face in each household.[24]

The national baseline survey was conducted between June 2011 and March 2012, having a total sample of 17,705 respondents. These respondents were followed up in 2013. The second national survey was finished in 2013, including a total of 18,605 respondents. In this study, we used the CHARLS longitudinal data from 2011 and 2013 waves.

### Measurements

#### Dependent variables

The dependent variable in our analysis was a categorically distributed variable with five discrete outcomes: public insurance only, private insurance only, double coverage of public and private health insurance, double coverage of both rural (NCMS) and urban public health insurance (URBMI or UEBMI) and no insurance.

#### Independent variables

The independent variables were carefully chosen based on the Andersen’s Behavioral Model of Health Services Use, a widely-used conceptual framework to investigate demand for health care and insurance [28]. We included variables of five dimensions: policy, health, socioeconomic, risk aversion, and other confounding factors.

- Policy-related factors: The policy factors reflected the influence of the residence registration system (Hukou) and health insurance policy on the demand for different health insurance. Variables included registration as rural or urban residents and migration status that are closely related to the regional health insurance policy. Migrants are defined as those who live in regions where they are not registered as local residence (Hukou), which may limit their access to local public health insurance and social welfare [13].
- Health-related factors: Health status was measured by self-reported health, chronic diseases, and respondents’ age. Self-reported health variable was divided into two groups: good health, and poor/fair health. Chronic diseases were measured by the question “have you been diagnosed with the following chronic conditions by a doctor: hypertension, dyslipidemia, diabetes or high blood sugar, cancer or malignant tumor, cardiovascular disease, stroke, chronic lung diseases, liver disease, kidney disease, stomach or other digestive disease, arthritis or rheumatism, asthma, emotional or psychiatric problems, memory-related disease”.
- Socioeconomic factors: Variables included household annual income per capita, educational attainment, and employment status. Income was divided into quintiles: the lowest, lower, middle, higher, and highest income groups. Respondents’ education levels were categorized into four groups: no formal education, not complete primary school but capable of reading/writing, completed primary school and junior high school and above. Employment status also contained four categories: farmers, unemployed or retired, informal, and formal employed.
- Risk aversion factors: Respondents’ risk aversion was measured by whether had health checkup during the past year and smoking status (never or past smokers vs. current smokers).
- Other confounding factors: These factors included gender, marital status (unmarried, widowed, separated or divorced vs. currently married), number of family members in the household, survey year and province-level fixed effects. Number of family members in the household was categorized into 3 groups: 1–2, 3–4, and 5 or more.

### Statistical analysis

Descriptive analyses were first performed to show the discrete outcomes of public, private health insurance and multiple insurance coverages, as well as the sociodemographic characteristics of the survey respondents. Using pooled data from the CHARLS 2011 and 2013, multinomial logit regression was performed to identify the determinants of public, private and multiple health insurance coverages. To determine whether the model meets the irrelevant alternative assumption (IIA) that the relative probability of choosing among two existing choices is unaffected by the addition or deletion of another alternatives, we performed the Hausman test [29]. The result of the test was insignificant and the coefficients did not change when we eliminated one of the categories, thus the IIA assumption was not violated. All regressions included province dummies to adjust for unobserved province-level fixed effects, and were adjusted for correlation at the individual level between the two panels.

We performed regression analysis with the total sample and separately with the rural and urban subsamples. We further estimated the association between public health insurance coverage and the demand for private health insurance. Relative Risk Ratios (RRRs) and Odds ratios (ORs) with 95% confidence intervals (CIs) were reported. Survey weights were applied to account for the multi-stage stratified sampling design in both descriptive and regression analyses. The sampling weights took into consideration of the representativeness of the estimates, and the household and individual non-response biases. All statistical analyses were performed using STATA 12.0 (College Station, TX: Stata Corp LP).

## Results

### Public, private and multiple health insurance coverage

Table 1 showed the public, private and multiple health insurance coverage among those aged 45 and over between 2011 and 2013 in China. In 2013, about 4.7% of them were uninsured, reducing from 7.4% in 2011. This decrease of the uninsured occurred partially due to an expansion of public health insurance from 91.3% in 2011 to 94.5% in 2013, and partly attributed to a boost of private health insurance from 7.2% in 2011 to 12.2% in 2013. Among those with double/multiple insurance coverage, around 11.4% had both public and private health insurance in 2013, almost doubled from that in 2011 (5.8%). Coverage by both rural (referred to NCMS) and urban public health insurance (referred to UEBMI or URBMI) slightly increased from 0.52% in 2011 to 0.70% in 2013.

**Table 1:** Public, private and multiple health insurance coverage among the population aged 45 and older in China, 2011–2013 CHARLS (%).

	2011 *			2013 *		
	Total	Rural ^a^	Urban ^a^	Total	Rural	Urban
Public health insurance only	84.91	87.65	78.03	82.40	86.19	73.38
Rural & urban public health insurance	0.52	0.55	0.45	0.70	0.68	0.72
Private health insurance only	1.36	0.47	3.64	0.86	0.37	1.97
Public & private health insurance	5.83	4.94	8.08	11.36	8.35	18.82
No health insurance	7.38	6.40	9.79	4.67	4.41	5.11
Total N	17,711	13,638	3,804	18,618	13,810	3,928

Note: All statistics adjusted for sampling weights.

^a^ Rural and urban was defined by the respondent’s registration status.

* The distribution was significantly different between rural and urban areas, P<0.001 calculated from Pearson Chi^2^ test.

We further compared the differences in health insurance coverage between rural and urban registered residents. We noticed that the percentage of people with public insurance only was significantly higher in registered rural residents than in those urban residents (P<0.001), whereas a higher percentage of urban residents purchased private health insurance and enjoyed coverage by both public and private health insurance in 2011 and 2013 panels.

### Characteristics of the survey respondents

Table 2 presented the descriptive statistics for the study population. Overall, the majority of the people (71.51% in 2011 and 71.23% in 2013) were registered rural residents. About 33.78% in 2011 and 27.98% in 2013 of the entire population were rural-to-urban migrants. Around 75% of the sample reported poor or fair health status, with around 65% of the population having at least one chronic condition. The average age was around 60 in both panels, and about half of the respondents were females. Around 85% were currently married and the average number of family members within a household was about 3.50. Average household income increased from 8930 RMB in 2011 to 10110 RMB in 2013. More than half of the sample had finished primary school education. And about 70% of the population in both panels was either self-employed farmers or unemployed/retired. Only less than 20% of the study sample was formally employed. A small proportion of people (18.06% in 2011 and 13.47% in 2013) had health checkup during the previous year and a slightly less than 30% of the sample were current smokers.

**Table 2:** Characteristics of the survey respondents, 2011–2013 CHARLS (%).

Characteristics	2011	2013
Rural residents	71.51	71.23
Migrant	33.78	27.98
Poor or fair health	74.06	75.58
Having any chronic disease	66.37	62.35
Age (years)*	59.20(10.15)	60.05(10.24)
Female	52.27	52.15
Currently Married	85.58	85.24
Number of family members in a household		
1–2	33.37	37.38
3–4	35.35	35.11
5 or more	31.28	27.51
Household income per capita (1000 Yuan)*	8.93(11.63)	10.11(17.29)
Education		
No formal education	25.61	24.42
Semi-literate but can read/write	16.65	16.49
Primary school	21.31	21.6
Junior high school and above	36.44	37.48
Employment status		
Farmers	37.65	37.07
Unemployed or retired	34.35	33.99
Informal employed	8.53	10.2
Formal employed	19.47	18.74
Health checkup during the past year	18.06	13.47
Currently smoking	29.97	29.80
N	17,711	18,618

*Mean, SD

Note: All statistics adjusted for sampling weights.

### Determinants of public, private and multiple health insurance coverage

Table 3 showed the determinants of health insurance coverage from the weighted multinomial logit regression. Each insurance outcome was compared to the base outcome of public insurance coverage only. As for the policy-related factors, compared to urban residents, rural registered population were significantly less likely to have both rural and urban public insurance (RRR = 0.52, 95% CI: 0.31–0.87), less likely to buy private insurance (RRR = 0.22, 95% CI: 0.16–0.31), less likely to have coverage by both public and private health insurance (RRR = 0.55, 95% CI: 0.48–0.62), and interestingly, also less likely to be uninsured (RRR = 0.40, 95% CI: 0.34–0.47). Generally, rural residents are expected to have more public insurance coverage as compared to other types of insurance. The likelihood of migrants being covered by multiple insurance, private insurance and being uninsured as compared to public insurance coverage were all significantly higher than local residents (P<0.05). As for the health-related factors, those with worse health status such as self-reported as poor or fair health and older people were less likely to purchase private insurance. They were also less likely to be uninsured as compared to public insurance coverage. Compared to males, females were less likely to be covered by both public and private health insurance (RRR = 0.89, 95% CI: 0.80–1.00).

**Table 3:** Determinants of public, private and multiple health insurance coverage: Results from the multinomial logit model (Base outcome: public insurance only).

	Multiple coverage: rural & urban public insurance	Private insurance only	Multiple coverage: public & private insurance	No insurance
Rural residents	0.51(0.30–0.87)**	0.22(0.16–0.31)***	0.55(0.48–0.62)***	0.40(0.34–0.46)***
Migrant	1.85(1.26–2.71)***	1.74(1.36–2.22)***	1.09(0.99–1.21)*	1.39(1.24–1.57)***
Poor or fair health	0.95(0.66–1.38)	0.64(0.49–0.82)**	0.85(0.77–0.94)***	0.89(0.79–1.01)*
Any chronic disease	1.55(1.08–2.23)**	0.88(0.69–1.12)	1.05(0.96–1.16)	0.68(0.61–0.77)***
Age (10 years)	0.85(0.68–1.06)	1.07(0.92–1.25)	0.82(0.77–0.87)***	0.82(0.76–0.88)***
Female	0.54(0.34–0.85)***	1.11(0.83–1.49)	0.89(0.80–1.00)*	1.06(0.92–1.22)
Currently Married	0.97(0.55–1.72)	1.13(0.77–1.64)	0.94(0.80–1.09)	0.52(0.45–0.60)***
Household size (referred to 1–2 family members)				
3–4	0.76(0.50–1.15)	1.02(0.78–1.35)	1.15(1.04–1.29)***	0.92(0.81–1.04)
5 or more	1.06(0.70–1.63)	0.82(0.59–1.14)	1.15(1.02–1.29)**	0.92(0.81–1.06)
Income (referred to lowest income)				
Lower	0.91(0.49–1.72)	0.77(0.44–1.35)	1.04(0.90–1.20)	0.78(0.68–0.90)***
Middle	1.49(0.84–2.62)	1.22(0.76–1.95)	1.02(0.88–1.17)	0.68(0.58–0.78)***
Higher	1.71(0.98–2.97)*	1.64(1.07–2.53)**	1.10(0.95–1.27)	0.68(0.58–0.78)***
Highest	1.61(0.87–2.97)	1.74(1.12–2.69)**	1.51(1.32–1.74)***	0.45(0.37–0.54)***
Education (referred to no formal education)				
No education but can read/write	0.58(0.29–1.14)	0.90(0.56–1.44)	0.83(0.71–0.97)**	0.80(0.69–0.94)***
Primary school	1.22(0.70–2.13)	0.84(0.54–1.30)	0.97(0.84–1.13)	0.65(0.55–0.76)***
Junior high school and above	1.38(0.76–2.53)	1.13(0.75–1.71)	1.23(1.07–1.42)***	0.47(0.40–0.56)***
Employment status (referred to farmers)				
Unemployed or retired	1.39(0.85–2.28)	1.75(1.19–2.58)***	0.99(0.88–1.12)	1.40(1.22–1.60)***
Informal employed	1.67(1.00–2.79)*	1.77(1.12–2.79)**	1.00(0.85–1.17)	1.90(1.61–2.25)***
Formal employed	1.76(1.10–2.81)**	1.05(0.69–1.60)	1.05(0.93–1.20)	1.24(1.05–1.47)**
Health checkup	1.68(1.16–2.42)***	0.97(0.71–1.32)	1.16(1.04–1.30)**	0.76(0.66–0.87)***
Currently smoking	0.78(0.53–1.16)	0.95(0.71–1.28)	0.88(0.79–0.99)**	1.08(0.94–1.23)
2013	1.81(1.33–2.44)***	0.55(0.43–0.69)***	2.11(1.95–2.29)***	0.59(0.54–0.65)***
Constant	0.01(0.00–0.12)***	0.04(0.01–0.20)***	0.54(0.27–1.08)*	0.98(0.27–3.61)
Pseudo R^2^	0.10			
Observations	35,068			

Notes: Relative Risk Ratios and 95% confidence intervals were shown. Multinomial logit model was used with public health insurance as the reference group. All models included sampling weights, province dummy and adjusted for clustering at the individual level. Significance level

*** p<0.01

** p<0.05

* p<0.10.

As expected, income and education levels were significantly associated with types of insurance coverage. If income level had increased from lowest to highest, the relative risk for private insurance purchase and coverage by both public and private insurance relative to public insurance only would be expected to increase by a factor of 1.74 (95% CI: 1.12–2.69) and 1.51 (95% CI: 1.32–1.74) respectively, whereas the relative risk for no insurance relative to public insurance only would be expected to decrease by a factor of 0.45 (95% CI: 0.37–0.54). A similar pattern was observed when education level had increased from no formal education to junior high school and above. Employment status was an important predictor of insurance coverage. Compared to farmers, the relative risk for coverage by both urban and rural insurance relative to public insurance only was significantly higher among those informally or formally employed, while they were also more likely to be uninsured (RRR = 1.90, 95% CI: 1.61–2.25 for informally employed; RRR = 1.24, 95% CI: 1.05–1.47 for formally employed). We also found evidence that having health checkups, an indicator of risk aversion, was positively associated with multiple insurance coverage, and negatively associated with being uninsured (P<0.001).

We further examined the determinants of health insurance coverage among rural and urban residents only (Table 4). The sample for one outcome “multiple coverage of rural & urban public insurance” was not large enough for analysis in the rural or urban subsamples, and was then excluded from these regressions. The results in the rural and urban subsamples were similar to those in the total sample. Among both subsamples, migrants were less likely to participate in public insurance, but were more likely to purchase private insurance. Compared to public insurance, the likelihood of urban residents purchasing only private insurance decreased significantly in 2013 compared to 2011 (RRR = 0.43, 95% CI: 0.32–0.58), but there was no significant change among the rural residents.

**Table 4:** Determinants of public, private and multiple health insurance coverage among rural/ urban residents: Results from the multinomial logit model (Base outcome: public insurance only).

	Rural residents			Urban residents		
	Private insurance only	Multiple coverage: public & private insurance	No insurance	Private insurance only	Multiple coverage: public & private insurance	No insurance
Migrant	1.39	1.13*	1.37***	2.32***	0.91	1.31**
	(0.91–2.12)	(1.00–1.27)	(1.19–1.58)	(1.70–3.16)	(0.75–1.11)	(1.04–1.64)
Poor or fair health	0.53***	0.80***	0.85**	0.70**	0.94	1.08
	(0.35–0.80)	(0.71–0.89)	(0.74–0.98)	(0.51–0.96)	(0.80–1.11)	(0.87–1.34)
Any chronic disease	0.99	1.00	0.75***	0.70**	1.08	0.55***
	(0.68–1.44)	(0.90–1.12)	(0.66–0.85)	(0.51–0.95)	(0.92–1.28)	(0.44–0.68)
Age (10 years)	0.83	0.74***	0.86***	1.30***	0.82***	0.75***
	(0.64–1.07)	(0.69–0.80)	(0.79–0.93)	(1.08–1.57)	(0.74–0.91)	(0.65–0.86)
Female	0.83	0.85**	1.12	1.44**	0.91	1.18
	(0.47–1.46)	(0.73–0.99)	(0.94–1.35)	(1.01–2.04)	(0.76–1.10)	(0.92–1.51)
Currently Married	1.33	0.95	0.50***	1.08	0.94	0.65***
	(0.65–2.73)	(0.79–1.13)	(0.43–0.59)	(0.69–1.71)	(0.72–1.22)	(0.48–0.86)
Household size (referred to 1–2 family members)							
3–4	0.90	1.09	0.88*	1.09	1.10	0.89
	(0.53–1.51)	(0.95–1.24)	(0.76–1.02)	(0.78–1.51)	(0.92–1.32)	(0.71–1.12)
5 or more	0.98	1.25***	0.88*	0.58**	1.07	0.82
	(0.61–1.57)	(1.09–1.43)	(0.76–1.02)	(0.36–0.95)	(0.86–1.33)	(0.62–1.08)
Income (referred to lowest income)						
Lower	0.85	1.05	0.83**	0.46	1.03	0.45***
	(0.45–1.63)	(0.90–1.22)	(0.71–0.96)	(0.13–1.63)	(0.64–1.67)	(0.30–0.68)
Middle	1.02	1.01	0.73***	1.19	0.85	0.46***
	(0.55–1.87)	(0.87–1.17)	(0.61–0.86)	(0.58–2.44)	(0.59–1.21)	(0.34–0.63)
Higher	1.59	1.04	0.71***	1.45	0.93	0.48***
	(0.87–2.93)	(0.88–1.22)	(0.59–0.85)	(0.78–2.70)	(0.68–1.28)	(0.37–0.63)
Highest	2.03**	1.33***	0.74***	1.47	1.36**	0.22***
	(1.08–3.83)	(1.13–1.58)	(0.60–0.91)	(0.80–2.70)	(1.01–1.84)	(0.16–0.29)
Education (referred to no formal education)						
No education but can read/write	0.70	0.76***	0.88	1.72	1.18	0.84
	(0.36–1.37)	(0.65–0.90)	(0.74–1.05)	(0.85–3.48)	(0.77–1.80)	(0.58–1.22)
Primary school	0.63	0.86*	0.77***	1.57	1.34	0.72*
	(0.34–1.17)	(0.74–1.01)	(0.64–0.92)	(0.81–3.02)	(0.91–1.98)	(0.51–1.02)
Junior high school and above	1.08	0.92	0.65***	1.76*	1.67***	0.45***
	(0.62–1.89)	(0.79–1.08)	(0.53–0.79)	(0.96–3.23)	(1.18–2.37)	(0.33–0.63)
Employment status (referred to farmers)						
Unemployed or retired	2.45***	1.06	1.59***	1.24	1.37*	1.00
	(1.51–3.96)	(0.92–1.21)	(1.37–1.84)	(0.67–2.32)	(0.98–1.91)	(0.71–1.39)
Informal employed	1.86*	0.93	1.69***	1.41	1.19	1.61**
	(1.00–3.45)	(0.78–1.12)	(1.38–2.06)	(0.69–2.90)	(0.80–1.77)	(1.09–2.39)
Formal employed	1.14	0.81***	1.27**	0.93	1.75***	0.82
	(0.65–1.99)	(0.70–0.94)	(1.05–1.54)	(0.48–1.80)	(1.24–2.47)	(0.55–1.23)
Health checkup	0.83	1.22***	0.76***	0.87	1.07	0.77**
	(0.48–1.43)	(1.06–1.39)	(0.64–0.91)	(0.60–1.27)	(0.89–1.28)	(0.61–0.96)
Currently smoking	0.74	0.97	1.10	1.19	0.90	1.16
	(0.44–1.23)	(0.84–1.12)	(0.93–1.30)	(0.83–1.71)	(0.74–1.09)	(0.91–1.49)
2013	0.77	1.79***	0.64***	0.43***	2.82***	0.49***
	(0.54–1.12)	(1.63–1.96)	(0.57–0.71)	(0.32–0.58)	(2.44–3.26)	(0.42–0.58)
Constant	0.01***	0.37***	0.41***	0.00***	0.12***	5.92***
	(0.00–0.14)	(0.22–0.64)	(0.21–0.80)	(0.00–0.02)	(0.04–0.31)	(1.86–18.89)
Pseudo R^2^	0.03			0.08		
Observations	27,352			7,716		

Notes: Relative Risk Ratios and 95% confidence intervals were shown. Multinomial logit model was used with public health insurance as the reference group. All models included sampling weights, province dummy and adjusted for clustering at the individual level. Significance level

*** p<0.01

** p<0.05

* p<0.10.

### Association between public insurance coverage and demand for private health insurance

Table 5 further presented the association between coverage by public health insurance, certain types of public insurance and demand for private insurance. We found that having any public health insurance (i.e. NCMS, URBMI or UEBMI) was expected to reduce the likelihood of purchasing private insurance, while keeping other variables constant in the model (OR = 0.55, 95% CI: 0.48–0.63), indicating a possible substitute effect between public and private health insurance. Further separating the sample by rural and urban residents, we found that this substitute effect was only significant for urban residents (OR = 0.33, 95% CI: 0.27–0.40), but not for rural residents (OR = 0.89, 95% CI: 0.71–1.11). While taking a closer look at the types of public health insurance, as compared to coverage by NCMS, having URBMI is negatively associated with demand for private insurance (OR = 0.77, 95% CI: 0.63–0.94). However, it should be noted that having UEBMI significantly increased the odds of purchasing private health insurance (OR = 1.33, 95% CI: 1.12–1.58) than covering by NCMS. And as expected, those without public insurance coverage were significantly more likely to purchase private insurance (OR = 1.91, 95% CI: 1.65–2.21).

**Table 5:** Association between public health insurance coverage, types of public insurance and purchase of private health insurance, results from the logit regressions.

Private health insurance	Total	Rural	Urban	Total
Having public health insurance	0.55(0.48–0.63)***	0.89(0.71–1.11)	0.33(0.27–0.40)***	
Types of public health insurance (referred to NCMS)				
Urban Resident Basic Medical Insurance (URBMI)				0.77(0.63–0.94)**
Urban Employee Basic Medical Insurance (UEBMI)				1.33(1.12–1.58)***
No public health insurance				1.91(1.65–2.21)***
Pseudo R^2^	0.10	0.11	0.08	0.10
Observations	35,314	27,522	7,784	35,314

Notes: Odds ratio and 95% confidence intervals were reported. Logit models were used, including all variables in Table 3. All models included sampling weights, province dummies, and adjusted for clustering at individual level. Significance level

*** p<0.01

** p<0.05

* p<0.10.

## Discussion

This study examined the determinants of public, private and multiple health insurance coverage among the population of retirement-age in China. Given the diversity of the population and substantial socioeconomic (SES) disparity in accessibility and affordability of health care among the Chinese elderly, this is the first study ever undertaken to comprehensively understand the status quo of China’s fragmented insurance system. Zhang and coauthor’s study used CHARLS 2011 and examined the predictors of being covered by UEBMI, URBMI, NCMS, or any insurance among urban and rural residents [26], while their study did not distinguish those covered by multiple insurance or those who purchased private insurance that were growing into an increasingly important component of China’s insurance system.

Our analysis showed that, till 2013, 94.5% of this population had at least one type of public insurance, and 12.2% purchased private insurance. In general, we found that (a) compared to urban residents, rural residents were more likely to participate in public health insurance. But rural-to-urban migrants were more likely to be uninsured. (b) Public health insurance coverage may crowd out private insurance market particularly among those urban residents who enrolled in URBMI. There exists a large SES disparity in both public and private insurance coverage.

The reason why rural residents had a higher percentage of public insurance coverage than urban residents might be related to the difference in enrollment unit between NCMS and the two urban insurance. The unit of enrollment for the NCMS is at the household level, but is at the individual level for UEBMI and URBMI [6, 30]. Due to the fact that NCMS achieved risk sharing among family members, large-size families with more elderly members may show greater willingness of insurance participation. However, for urban families, people with employment can be covered by UEBMI and other dwellers without formal employment can only enroll in URBMI. Therefore, household members cannot share risks in urban residents. Allowing household coverage in the urban insurance schemes might help to achieve universal coverage among the urban residents. [31]

It is worth noting that migration may reduce the likelihood of being covered by public health insurance, but increase the likelihood of multiple coverage. Although the average age of the migrants was around 35 years, the first generation of the migrants has been turning to their 50s since 1980s. In our national sample, migrants accounted for about 30% of the entire population aged 45 and older. With a higher risk for developing chronic diseases, they are in greater need of health insurance coverage than the younger ones. However, a national survey showed that in 2014, the older migrants aged 45 and 60 had similar public insurance coverage with those aged 15–45 years (75.6% vs 75.0%), but had slightly lower private insurance coverage compared with the younger ones (3.9% vs 4.6%) [26]. One explanation to this low coverage was that many of the migrants were employed in informal sectors or small businesses which made them not qualified for UEBMI enrollment [14]. Another explanation was that public insurance, administered at the county or municipal level, usually did not cover health services outside of their local regions. Therefore, migrants had less incentive to participate in public insurance due to little opportunity for reimbursement. In addition, to gain insurance reimbursement from their destination place, migrants who were covered by rural insurance were more likely to also participate in urban public health insurance. But this dual coverage would increase overall premiums for the enrollees and administrative costs for the government agencies. Since 2011, some regions launched pilot policies to integrate NCMS and URBMI into one scheme, which may expand urban insurance coverage for the rural-to-urban migrant workers [15]. However, the lack of integration across regions in the insurance system and the rigid *Hukou* system would continuously be barriers for rural migrant workers to access urban health care [14]. Thus, the integration of rural and urban public health insurance across regions and the management of both insurance by one government agency would be a better policy solution in the foreseeable future.

Understanding the role of private insurance is a little difficult since private insurance can supplement public insurance for China to achieve universal health coverage [19], but it can also be substituted by the expansion of public insurance [32]. Both evidence has been observed in our data and in other countries [22, 32–36]. On the one hand, our results showed that UEBMI enrollees and high SES population were more likely to buy private insurance. A China-based study also found that high-income adults were more likely to purchase private insurance when they were covered by NCMS, but for low-income people, the likelihood of buying private insurance decreased with public insurance coverage [22]. On the other hand, our findings suggested that public insurance coverage was associated with a reduced demand for private insurance, especially for urban residents who were covered by URBMI. This was consistent with a US-based study that found public insurance, subsidy or compensation was associated with a lower likelihood of private insurance purchase among the Medicaid enrollees [36]. However, we did not observe an adverse selection in the demand for private insurance, a phenomenon also documented in Liu and coauthor’s study [22]. There seemed counterintuitive that the people aged 45 and older with poor or fair health had a lower likelihood of purchasing private insurance. Other studies have observed that risk-takers are less likely to maintain good health and buy health insurance [37]. But these results may be understood in the China-specific context. First, health insurance is relatively new to Chinese residents particularly for the rural poor. The high coverage of public insurance was achieved due to the compulsory or semi- compulsory nature of the insurance [38]. But private insurance was much less familiar to the elderly population who received no or limited education. The complex design in the private insurance plans prohibited those who needed insurance but were lacking the cognitive ability to understand the details [38–40]. These double roles private insurance played in China’s health insurance system may exacerbate the SES inequality in access to health insurance among China’s middle-aged and elderly population.

There are several limitations in our analysis. First, albeit at the national level, the CHARLS sample only represented the population aged 45 and older in mainland China, except for residents of Tibet. The results can hardly be generalizable to the entire population in China. In particular, the majority of the migrant workers are younger adults, and their health status and demand for insurance could be different from the older adults [41]. Second, we cannot rule out the possibility of misspecification due to self-report bias. For instance, some people may have double rural or urban public insurance coverage–one was obtained from their hometown and the other was offered by the places where they live and work, which may lead to underestimation of the multiple insurance coverage. Third, our sample does not contain information on usual source of care, thus we cannot estimate whether access to health care influenced the take-up of health insurance. However, we expect this bias to be minimal. After the health care reform in 2009, access to health care has been largely improved, and most people can go to a health care clinic/ provider within 15 minutes [9]. In addition, access to health care (such as distance) mainly differs by region, and in our regression analysis, we have controlled for regional variation such as rural/urban areas and provinces. The health checkup variable was also a good proxy for access to care, which was included in the regression.

In conclusion, although China’s public insurance schemes have gradually covered the majority of its population in rural and urban areas, the domestic migrants, the poor and the vulnerable remained in the edge of the system. The growing private insurance market did not provide sufficient financial protection and was not accessible for people with the greatest need. With the rapid urbanization and population ageing, China needs to achieve universal coverage as well as reduce SES disparity in access to health insurance. Chinese government should reform the current fragmented insurance system by integrating the urban and rural public insurance schemes across regions and making them managed by one government agency, and removing the barriers for the middle-income and low-income to access private insurance. Such efforts would require a strong partnership across regional and national public sectors, and trust between public and private sectors [19]. Future research will focus on the geographic variation and the SES disparity in insurance integration and coverage, as well as its changes to cover vulnerable groups including those with low SES and chronic diseases.
